# Structural characterization of site-modified nanocapsid with monodispersed gold clusters

**DOI:** 10.1038/s41598-017-17171-x

**Published:** 2017-12-06

**Authors:** Marie C Stark, Mo A Baikoghli, Tanja Lahtinen, Sami Malola, Li Xing, Michelle Nguyen, Marina Nguyen, Aria Sikaroudi, Varpu Marjomäki, Hannu Häkkinen, R Holland Cheng

**Affiliations:** 1 Department of Biology and Environmental Science, Nanoscience center, University of Jyväskylä, Jyväskylä, FI-40014 Finland; 2 Department of Molecular and Cellular Biology, University of California, Davis, CA 95616 USA; 3 Department of Chemistry, Nanoscience Center, University of Jyväskylä, Jyväskylä, FI-40014 Finland; 4 Department of Physics, Nanoscience center, University of Jyväskylä, Jyväskylä, FI-40014 Finland

## Abstract

Hepatitis E Virus-like particles self-assemble in to noninfectious nanocapsids that are resistant to proteolytic/acidic mucosal delivery conditions. Previously, the nanocapsid was engineered to specifically bind and enter breast cancer cells, where successful tumor targeting was demonstrated in animal models. In the present study, the nanocapsid surface was modified with a solvent-exposed cysteine to conjugate monolayer protected gold nanoclusters (AuNC). Unlike commercially available gold nanoparticles, AuNCs monodisperse in water and are composed of a discrete number of gold atoms, forming a crystalline gold core. Au_102_ *p*MBA_44_ (Au_102_) was an ideal conjugate given its small 2.5 nm size and detectability in cryoEM. Au_102_ was bound directly to nanocapsid surface cysteines via direct ligand exchange. In addition, Au_102_ was functionalized with a maleimide linker (Au_102__C_6_MI) for maleimide-thiol conjugation to nanocapsid cysteines. The AuNC-bound nanocapsid constructs were conjugated in various conditions. We found Au_102__C_6_MI to bind nanocapsid more efficiently, while Au_102_ remained more soluble over time. Nanocapsids conjugated to Au_102__C_6_MI were imaged in cryoEM for single particle reconstruction to localize AuNC position on the nanocapsid surface. We resolved five unique high intensity volumes that formed a ring-shaped density at the 5-fold symmetry center. This finding was further supported by independent rigid modeling.

## Introduction

Biological constructs with multiple axes of symmetry, such as viruses, are widely used as scaffolds to orient molecules in a uniform arrangement^1,2^. Chemical conjugation is an efficient method to selectively bind naturally occurring and synthetic molecules to a biological complex. The structural protein of Hepaptis E Virus (HEV) self assembles in to a highly stable icosahedral capsid. Because HEV is a fecal-orally transmitted virus, assembled HEV capsid protein (HEV nanocapsid) can withstand harsh proteolytic and acidic oral delivery conditions^3,4^. Previously, fluorescently labeled HEV nanocapsids bound to tumor-specific peptides were shown to target tumor tissue in animal models^5,6^. This proof-of-concept study demonstrated the diagnostic potential of chemically modified HEV nanocapsids.

In the present study, virus-like particles of HEV nanocapsids engineered with a surface-exposed cysteine were bound to molecule-like gold nanoclusters. Gold nanoparticles (AuNPs) are universal labeling tools for biological imaging due to their low toxicity, high solubility, contrast properties, and unique light absorbance spectra^7,8^. AuNPs function as contrast agents in electron microscopy (EM) as their electron-dense gold core generates high phase and amplitude contrast, distinguishing AuNPs from biological material. Some AuNPs have plasmonic and near-infrared absorbance (NIR), a well-described diagnostic imaging signal detectable in deep-tissue samples and *in vivo* ^7–11^. Therapeutic applications to localize the photo-thermal effect of AuNPs are also being explored^7,9,12,13^. Monolayer-protected gold nanoclusters (AuNCs) are distinct from traditional AuNPs such as colloidal AuNPs and Nanogold^®^. At a nanoscale, solubilized colloidal gold is relatively large, heterogeneous in solution and either non-specifically binds biomaterial or requires extended functionalized linkers, limiting the achievable resolution in EM imaging^14,15^.

This work reports the first successful Murray place exchange conjugation of AuNCs to a large icosahedral complex. In earlier studies, AuNC was indirectly localized to a N9 neuraminidase protomer through an intermediate Fab fragment conjugated to AuNC^16^. These AuNC-protein complexes were imaged in EM and structurally characterized with single particle reconstruction. The reconstruction results from this work demonstrated the rigidity of protein-bound AuNCs and suggested larger macromolecules could bind AuNCs functionalized with short linkers.

In the present study, we resolved the structure of a large icosahedral complex bound to AuNCs functionalized with short linkers. The AuNC used in this study, Au_102_ *p*MBA_44_ (Au_102_), has a 1.5 nm gold core composed of 102 gold atoms. Protected by 44 thiolate ligands, *p*-mercaptobenzoic acid (*p*MBA), Au_102_ is about 2.5 nm in diameter. Properties of Au_102_ such as structure, surface charge distribution, and deprotonation effects in water have been extensively characterized^17–20^. Like other thiol-protected AuNCs, Au_102_ is monodispersed in water and exhibits a distinct light absorbance profile^20–22^. Previously, Au_102_ was functionalized with a thiol-reactive linker, *N-*(6-hydroxyhexyl)maleimide (Au_102__C_6_MI), to bind native enteroviruses cysteines^23^. Because native cysteine residues were located just below capsid surface, linker-functionalization was necessary to access the enterovirus cysteines. Enteroviruses bound Au_102__C_6_MI without impairing virus infection, but a two-day incubation at 37 °C was necessary to bind available cysteine sites. These enterovirus conjugation studies provided the ground work for AuNC conjugation to surface-engineered nanocapsid.

**Figure 1:**
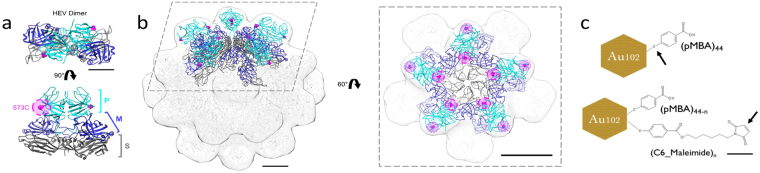
Schematic for nanocapsid and AuNC cysteine conjugation. (**a**) X-Ray cystral structure of nanocapsid dimer highlighting 573 C binding site on the nancapsid P-domain. (**b**) When dimer building blocks associate to form a pentamer, 573 C sites faces the 5-fold center. The crystal structure of this pentamer was docked over a surface rendition of the full T = 1 nanocapsid crystal structure^36^. (**c**) The cysteine-reactive sites on AuNC constructs^23^ are depicted with a black arrow, illustrating cysteine replacement site for Au_102_ (top) and the maleimide-thiol coupling site in Au_102__C_6_MI (bottom).

The current study aims to investigate conjugation efficiency and the 3D structure of AuNC-bound nanocapsids. Native HEV lacks surface exposed cysteine residues, so conjugation was limited to the engineered cysteine sites. This site-specific conjugation enabled more precise tuning of the nanocapsid surface. Au_102_ was bound to nanocapsids through direct ligand exchange while Au_102__C_6_MI was bound via maleimide-thiol conjugation. Nanocapsids self-assemble to form an empty T = 1 icosahedral capsid composed of a S, M, and P domain (Fig. 1a). The S and M domains provide an icosahedral base from which the surface P-domain extends from a proline-rich flexible hinge^24,25^. The P domain forms a spike protruding away from the capsid shell, stabilizing P domain surface interactions such as chemical conjugation. After conjugation efficiency and stability assessment, AuNC-bound nanocapsids were imaged for 3D structure evaluation. Using cryoEM data from Au_102__C_6_MI-bound nanocapsids, a 3D density map of AuNC-bound nanocapsids was resolved through single particle reconstruction (SPR). Conjugation efficiency and structural analysis are critical to the development of a stable nanocapsid delivery platform.

## Results

Previously, five cysteine replacement sites were evaluated based on surface exposure, structural flexibility, as well as epitope antigenicity and cell-binding^6^. Based on previous research^6,26^ and preliminary screening of AuNC conjugation to various constructs, we selected HEV nanocapsid 573 C to bind AuNCs. 573 C is located on the outer edge of the nanocapsid dimer, just below the upper tip, such that five 573 C sites face the 5-fold symmetry center (Fig. 1b). The proximity of 573 C to the 5-fold cavity exposes cysteine sites to bind contrast molecules, but leaves the top of the dimer surface available for additional cell-targeting modulation. Nanocapsids were conjugated to Au_102_ and Au_102__C_6_MI via ligand exchange and maleimide-thiol coupling, respectively (Fig. 1c).

### Gold Nanocluster Conjugation

**Figure 2:**
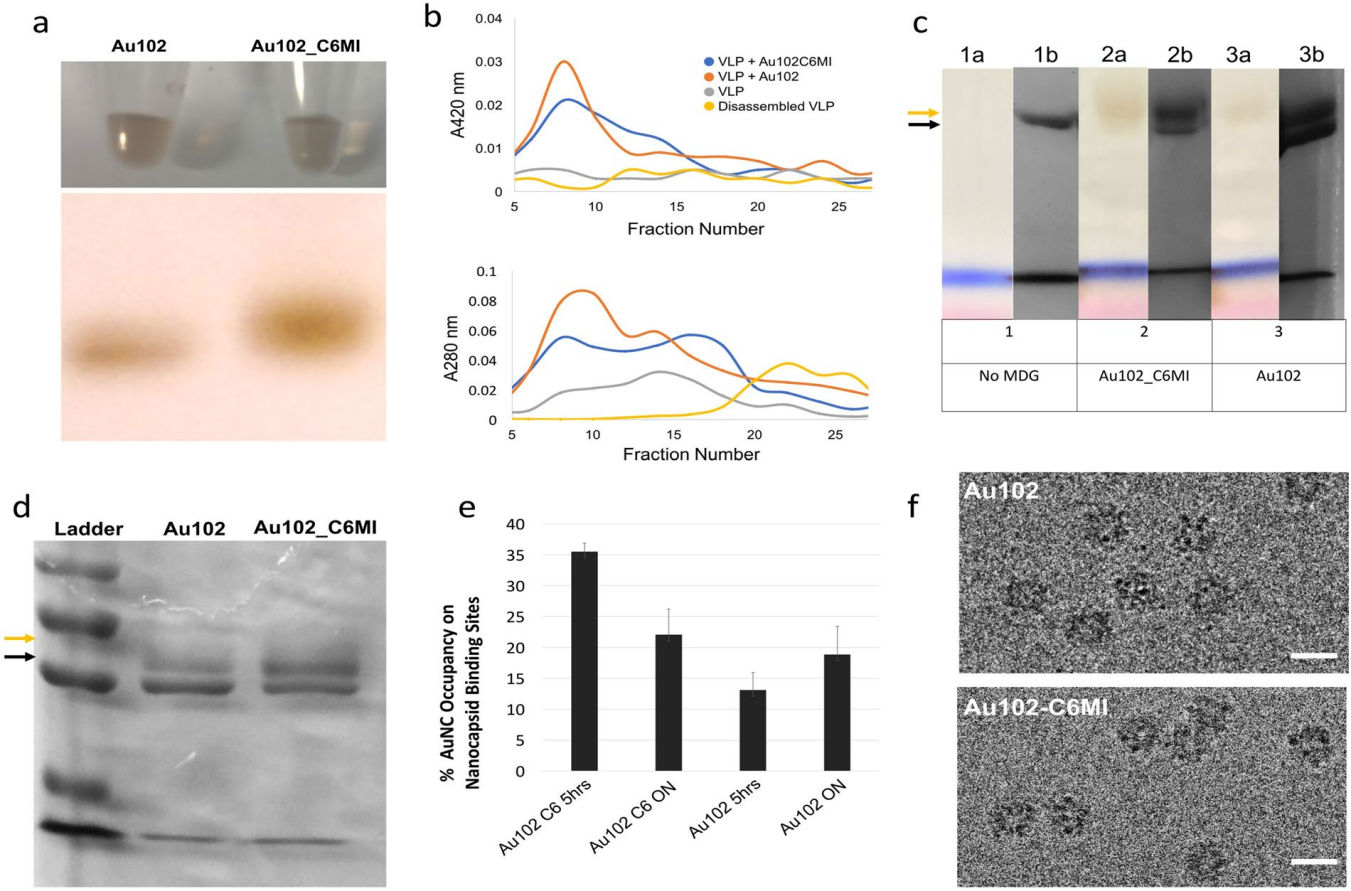
Characterization of AuNC and screening for conjugation to nanocapsid. (**a**) AuNCs solubilized in neutral buffer and visualized in plastic micro tubes (top) and run through native PAGE (bottom). (**b**) A_280nm_ (top) and A_420nm_ (bottom) nanodrop readings for size exclusion fractions of AuNC-bound and unbound nanocapsids. (**c**) Non-reducing SDS-PAGE before (1a–3a) and after (1b–3b) coomassie blue staining. (**c,d**) Non-reducing SDS PAGE gels of AuNC-bound nanocapsids samples bound for ON (c) and 5 h (**d**) where black arrows indicate to nanocapsid banding (~52 kDa) and gold arrows indicate AuNC-bound nanocapsid banding (~56 kDa). (**e**) Au_102_ occupancy on nanocapsid surface. Percent occupancy determined molar ratio of (AuNC): (nanocapsid binding sites) based on UV-vis as described in Equation 1. (**f**) CryoEM images of AuNC-bound nanocapsids.

Purified AuNCs, Au_102_ and Au_102__C_6_MI, were both solubilized in sterile ddH_2_0. Gel electrophoresis of water solubilized AuNCs confirmed the adequate size and homogeneity of the gold clusters^15,27^ as depicted in Fig. 2a (full gel in Figure S1a). Confirmation of the functionalization with Au_102__C_6_MI was suggested by smeared banding in polyacrylamide gel^23^. The distinctive brown color of AuNCs, arising from the size of the gold core, remained unchanged in the C_6_MI functionalized Au_102_^15,22^. AuNCs were imaged with EM to confirm the expected cluster diameter and uniform size coinciding with previous imaging results^28^.

Nanocapsids were bound to AuNCs at RT and samples were passed through a column containing size exclusion chromatography resin for fraction collection. As a crude size exclusion method, this procedure only effectively removes small, unbound AuNCs which remain in column resin. The large size of nanocapsids was beyond the resolvable limit of the resin used, which tended to result in a broad elution curve. However, there was a consistent elution profile where disassembled nanocapsids eluted in later fractions than assembled nanocapsids and unbound nanocapsids eluted in later fractions than AuNC-bound nanocapsids (Fig. 2b). Spectrophotometry measurements at absorbance values of 280 nm and 420 nm wavelengths (A_280nm_ and A_420nm_) were acquired for each fraction. As pure protein, unbound nanocapsids absorbance signal peaks at A_280nm_ but do not absorb 420 nm light. AuNCs have been shown to absorb light across a broad UV-vis spectrum, with particularly high 280 nm light absorbance^15,19^. Using Au_102_-specific extinction coefficients^20^, A_420nm_ values were measured to determine the concentration of AuNC in a sample^14,20^. For a nanocapsid + AuNC sample, A_280nm_ measurements alone could not be used to determine protein concentration. Instead, the A_420nm_ measurement was used to infer the expected A_280nm_ absorbance for AuNC without protein. The expected A_280nm_ value was subtracted from the measured A_280nm_ value to determine protein concentration (see Equation 1 in Experimental Procedures). From fractions with detectable A_420nm_ values, often accompanied by a visible yellow-brown color in solution, the presence of nanocapsid protein was confirmed by SDS-PAGE. TEM imaging confirmed the icosahedral structure of nanocapsid remained intact.

Assembled nanocapsids, disassembled nanocapsids, AuNC, and nanocapsids bound to AuNC for 24 h were run through size exclusion columns for fraction collection. Unbound AuNC remained in the column (Figure S2a) and absorbance measurements were acquired for the other sample fractions (Fig. 2b). A_280nm_ for AuNC-bound nanocapsid was significantly higher than that of assembled and disassembled nanocapsids, which had no detectable A_420nm_ signal. Disassembled nanocapsids eluted in later fractions than assembled nanocapsids peaking at A_280nm_ = 0.038 and A_280nm_ = 0.024, respectively. A_280nm_ and A_420nm_ values were slightly higher for Au_102__C_6_MI-bound nanocapsids than Au_102_-bound nanocapsids, peaking at A_280nm_ = 0.085 and A_280nm_ = 0.057, respectively.

Following overnight conjugation, Au_102__C_6_MI partially precipitated in solution while Au_102_ remained fully soluble. For Au_102_ conjugation, pre-incubating the nanocapsid with TCEP (tris(2-carboxyethyl)phosphine) resulted in an increased molar ratio of bound AuNCs to available binding sites (TCEP-free data not shown). Non-reducing SDS-PAGE (Fig. 2C) was run to confirm the presence of two distinct protein bands: an unbound, 52 kDa nanocapsid subunit and a ~56 kDa AuNC-bound subunit (full gel in Figure S1b). Prior to coomassie staining of gels, a coffee colored band was apparent around 56 kDa. Both the nanocapsid and AuNC-bound nanocapsid bands were visible after coomassie blue staining. 5 h conjugation samples were also run through size exclusion chromatography (Figure S2b) and in SDS-PAGE (Fig. 2d) suggesting Au_102__C6MI binding efficiency was higher than that of Au_102_ over shorter conjugation time. After 5hr and ON conjugation, protein and AuNC concentration were calculated using previously described concentration calculations^14,20^ (Fig. 2e) and summarized in Equation 1. Among peak A420 nm fractions for Au_102_, average binding occupancy was 13.1% and 18.9% after 5 hour and overnight conjugation, respectively, while Au_102_C_6_MI average binding occupancy was 35.5% and 22.1%, respectively.

Nanocapsids conjugated to AuNC were imaged in cryoEM (Fig. 2f). For observation purposes in cryoEM, optimal defocus values were distinct for nanocapsid protein and AuNC. Nanocapsids conjugated to AuNCs in Tris-NaCl pH = 7.5 appeared linked together in cryoEM, while nanocapsids conjugated to AuNC in MES pH = 6.5 were better dispersed (Figure S2c). Freshly prepared AuNCs and AuNCs prepared several months earlier were solubilized in MES and Tris-NaCl. Initial concentration was determined with A_420_ nm measurement followed by a second A_420_ nm measurement after one day and one week in solution (Figure S2d). These results suggested, particularly for older AuNC preparations, Au_102__C_6_MI exhibited the most significant precipitation in Tris-NaCl whereas Au_102_ remained soluble in all buffer conditions.

### Rigid Modeling of AuNC Position on Nanocapsid 5-fold

**Figure 3:**
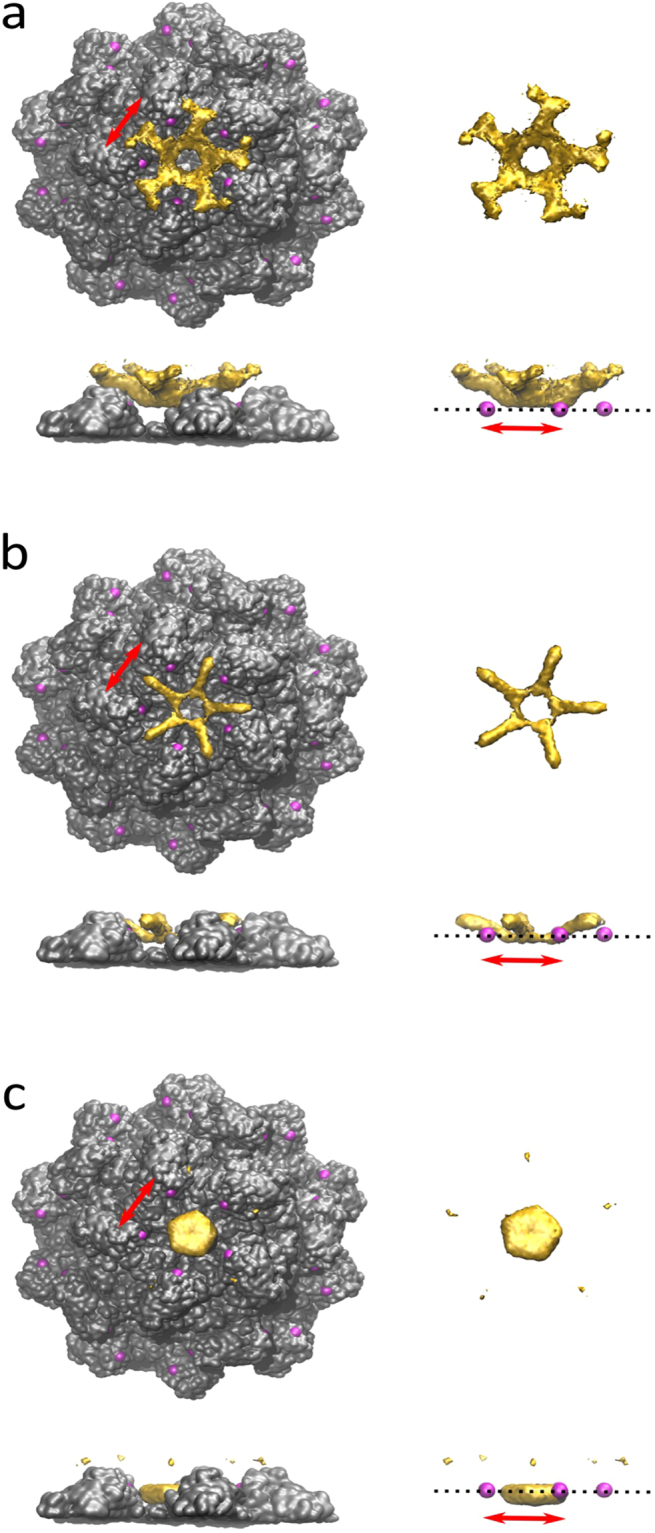
Rigid modeling of Au_102__C_6_MI conjugation to the nanocapsid binding site. Three rigid models illustrate probable positions of AuNCs on the nanocapsid surface within specified distance parameters determined by Au_102__C_6_MI atomic structure and the cysteine binding site. The density of AuNCs (colored gold) was built on the surface of nanocapsid crystal structure (colored gray) by adding the modeled electron density of Au_102_ at each accessible position with a specified distance from the binding site (pink). (**a–c**) The left image shows nanocapsids with modeled AuNC densities and the AuNC densities alone on the right. The upper image is a top view of the 5-fold symmetry center while the bottom shows a side view of the 5-fold symmetry center. Probe radii distance and binding site to probe center distance parameters for each model were as follows: (**a**) 1.6–3.0 nm and <3.0 nm; (**b**) 1.6–2.6 nm and 2.1–3.7 nm; (**c**) 1.6–2.9 nm and <4.2 nm, respectively. Red arrow scale bar is 4.5 nm.

To model where AuNC densities could localize on the nanocapsid surface, rigid modeling of AuNC-bound nanocapsid was performed independent of the 3D reconstruction results (Fig. 3). The model described probable accessible positions for AuNC densities on the nanocapsid surface. The parameters of this model were based on length of C_6_MI linker, the nanocapsid binding site (573C), nanocapsid x-ray crystal structure^29^, and the radial density of Au_102_. The radial density of Au_102_ was constructed from the coordinates of atoms weighted by the atomic number, normalization to a spherical area, and Gaussian broadening. Accessible positions on the nanocapsid surface were illustrated using spherical probes of specified size range representing the effective size of Au_102__C_6_MI. The density of AuNCs (colored gold) was built on the surface of nanocapsid crystal structure (colored gray) by adding the modeled electron density of Au_102_ at each accessible position. This modelling assumed each position of Au_102__C_6_MI had equal probability and restricted spherical probe densities from overlapping with nanocapsid coordinates within van der Waals radii of atoms. Positions for the AuNCs were allowed within a specified distance range from the N573C binding site contingent on the length of the *p*MBA surface plus the C_6_MI linker. Three separate distance parameters were implemented for rigid modeling (Fig. 3a–c) as follows: probe radii distance and binding site to probe center distance (3a) 1.6–3.0 nm and <3.0 nm; (3b) 1.6–2.6 nm and 2.1–3.7 nm; (3c) 1.6–2.9 nm and <4.2 nm, respectively.

In the first model (Fig. 3a), the maximum distance parameter between binding site to probe center closely resembled the atomic length Au_102__C_6_MI measured from maleimide end to gold core. With parameters that matched static atomic structures, the first model assumed little to no nanocapsid structural change. Potential structural changes, particularly to the P-domain, upon AuNC conjugation, were also considered. To account for the possible movement of the cysteine binding site upon conjugation, we also modeled slightly increased distance ranges between binding site and gold core in Fig. 3b, with the most extreme range shown in Fig. 3c. The rigid modeling suggested AuNC likely localized near the nanocapsid 5-fold cavity, forming a ring-like structure around the 5-fold symmetry center.

### 3D Reconstruction of AuNC Conjugated Nanocapsid

**Figure 4:**
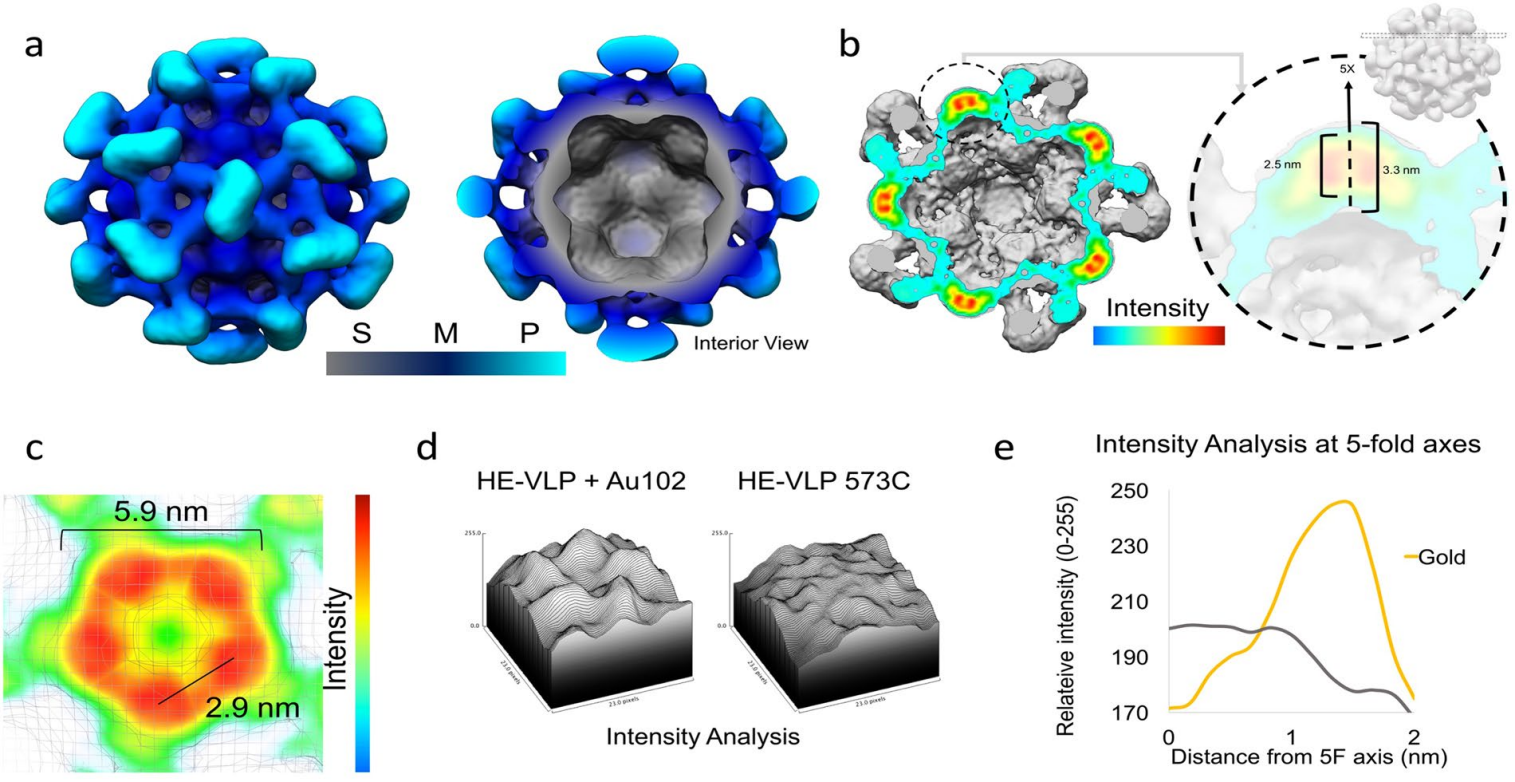
3-D structure of nanocapsid T = 1, conjugated to AuNC (**a**) Surface rendering of 3D reconstructed volumetric map of nanocapsid bound to AuNC, depicting both the outer surface (left) and capsid interior (right). The shell, middle, and protrusion domains are radially colored with gray, dark blue, and cyan, respectively. Scale bar is 6.0 nm. (**b**) Non-orthogonal intensity slice analysis around 5-fold axis showing high intensity regions associated with AuNC. Overall diameter of each AuNC measured to be 2.5 nm and distance from bottom to top of 5-fold measured to be 3.3 nm. (**c**) Plane intensity analysis showing distinct high intensity regions with a total diameter of 5.9 nm and distance between each AuNC 2.9 nm. (**d**) Quantitative comparison of surface intensity around the 5-fold axis between nanocapsid with (left) and without AuNC (right). Nanocapsid-bound AuNC showing distinct peaks around the 5-fold, not observed in the no-AuNC map. (**e**) Quantitative 1D intensity histogram comparing nanocapsid-bound AuNC (gold line) with nanocapsid (gray). Intensity peak ~1.8 nm away from the center of the 5-fold, in good agreement with observed position of AuNC.

CryoEM micrographs of Au_102__C_6_MI-bound nanocapsids were collected to generate a data set for Single Particle Reconstruction (SPR). Semi-automated particle selection methods were used to select a total of 5,276 particles for refinement and reconstruction. Iterative de novo initial models were generated to determine and cross-validate the particle parameters^30^. To optimize the 2D alignment and orientation during refinement, a polar Fourier transform (PFT) method with radial range pushed to 10 Å beyond the particle edge was implemented in conjunction with center search^31–33^. 3D reconstruction methods were performed with imposed icosahedral symmetry and raw data is shown in Figure S3. In the resulting icosahedral density map, the S, M, and P domains of the nanocapsid were clearly resolved (Fig. 4a). In addition to reconstructing distinct features of a T = 1 HEV nanocapsid, five unique high-intensity regions were identified around the nanocapsid five-fold and localized to twelve icosahedral vertices. For validation purposes, 3D reconstruction of unbound nanocapsid was performed in parallel with AuNC-bound nanocapsid (Figure S4). Both density maps manifested the same structural S, M, and P domain features, except the unbound nanocapsid map did not display a high-intensity 5-fold volume.

Local intensity analysis indicated the high intensity regions were distinguishable from the protein, forming a ring-like cluster around the nanocapsid 5-fold axes. These intensities were observed slightly above the S domain around the 5-fold axes. In the 3D reconstruction, the ring-like cluster that was 6 nm in diameter was comprised of five individual densities, each 2.5 nm in diameter (Fig. 4b,c). The distance between the center a given AuNC density and the nearest 573 C binding site was about 2–3 nm. The localization of unique AuNC densities was validated with difference mapping between the resolved structures of AuNC-bound nanocapsid and unbound nanocapsid (Figure S4) as well as intensity analysis (Fig. 4d,e). Local intensity profiling characterized the difference in intensity peaks around the 5-fold. The AuNC-bound map displayed distinctive high intensity peaks corresponding to the ring-like distribution of AuNCs, slightly above the S domain density (Fig. 4d). These intensities were quantified in a 1D graph to assess the local intensity differences between AuNC-bound nanocapsid and native nanocapsid (Fig. 4e).

**Figure 5:**
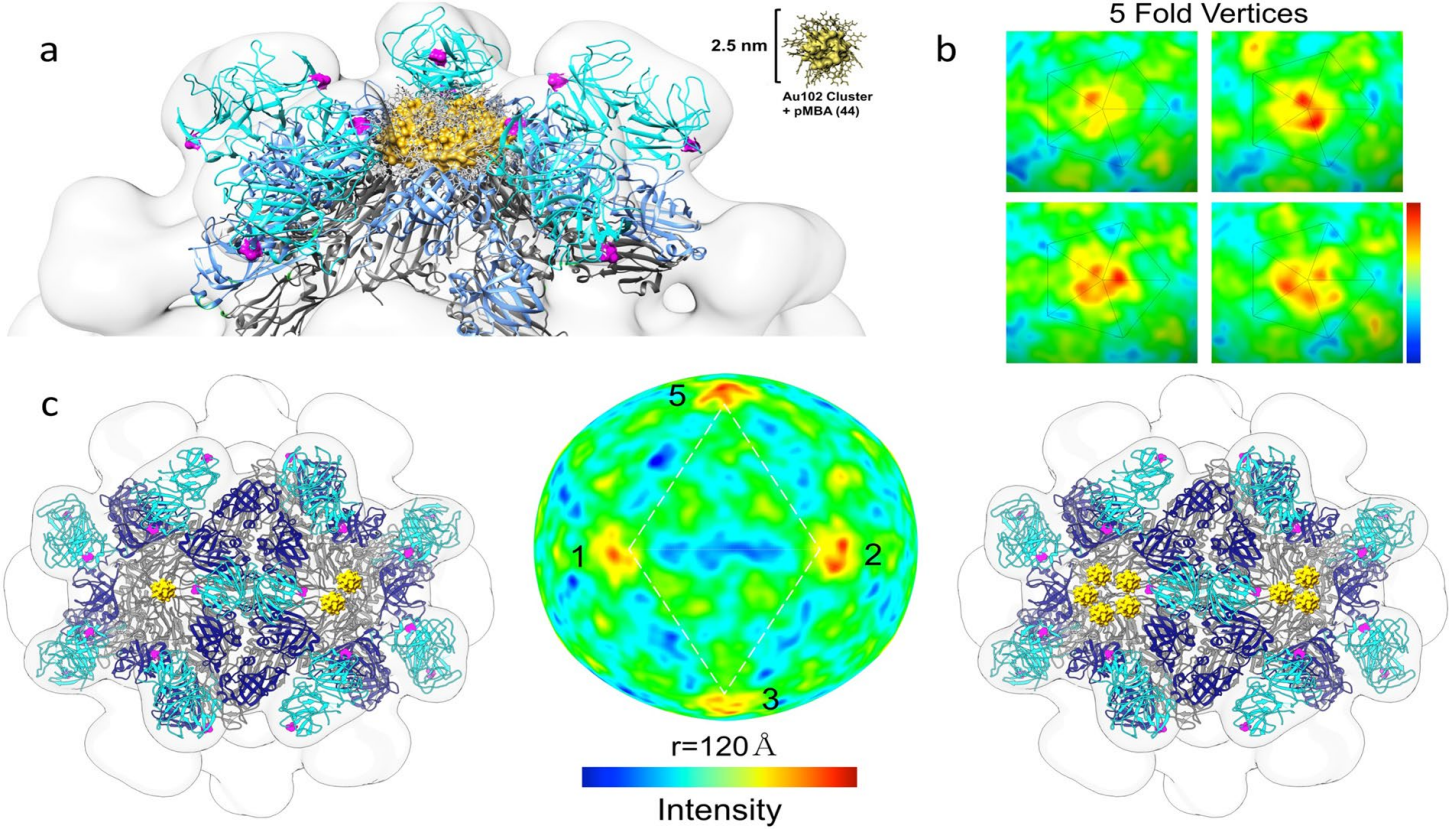
3D reconstruction of AuNC-bound nanocapsid based on relaxed symmetry TEM analysis. (**a**) 3D modeling of AuNC-bound nanocapsid with 5 AuNC clusters arranged around the 5-fold axis in a ring-shaped array. (**b**) Following relaxed symmetry 3D-refinement of AuNC-bound nanocapsid, 5-fold vertices show an uneven distribution of AuNC from the relaxed symmetry reconstruction. These vertices are colored based on slice intensity analysis with 5-fold vertices having between 0–5 intensities attributable to AuNCs. (**c**) Illustration of various scenarios based on relaxed symmetry TEM 3D reconstruction results. Radial cueing 120 Å away from the nanocapsid origin.

To visualize AuNC conjugation, we modeled Au_102_ crystal structures^19^ on the nanocapsid surface based on the 5-fold AuNC densities resolved in our 3D reconstruction (Fig. 5a). 3D reconstruction with icosahedral symmetry will generate symmetric AuNC densities at each 5-fold, and therefore appear as 100% AuNC binding. However, 100% AuNC conjugation is unlikely and was not supported by the spectrophotometric and SDS-PAGE data. To assess the relative density distribution at the 5-fold without imposed icosahedral symmetry, we implemented relaxed symmetry into reconstruction refinement. Of high intensity volumes resolved in the relaxed symmetry structure, the relative position of 5-fold densities matched that of the imposed symmetry structure. Radial cueing analysis showed a heterogeneous distribution of intensity peaks around the 12 nanocapsid 5-fold vertices (Fig. 5b). Heterogeneity in 5-fold intensities indicated between one to five AuNCs bound cysteines at each 5-fold. According to the relaxed symmetry reconstruction, of those highly bound nanocapsids, between 40–70% of binding sites were occupied, with majority of 5-fold axes bound to one or more AuNC. Based on these findings, we modeled possible scenarios that would reflect the resulting AuNC distribution around the 5-fold (Fig. 5c).

## Discussion

The higher conjugation efficiency of Au_102__C_6_MI over short incubation time as shown in Fig. 2d was most likely attributed to the increased binding site accessibility of an extended C_6_MI linker. Though overnight conjugation increased Au_102_ binding efficiency, the observed reduction in conjugation efficiency of Au_102__C_6_MI over time may be a consequence of precipitation. Au_102__C_6_MI tended to precipitate in solution over time, particularly at high pH (Figure S2d). In addition, maleimide ring hydrolysis causing the ring to open and lose thiol reactivity will reduce binding efficiency^34^. The hydrophobicity of the C_6_MI linker may have induced hydrophobic surface interactions between functionalized AuNCs, contributing to Au_102__C_6_MI precipitation in solution.

Rigid modeling of AuNC-bound nanocapsids revealed a localization of AuNC densities around the 5-fold symmetry center. In the first two models (Fig. 3a,b), gold densities form a ring of AuNC densities similar to that observed in our 3D reconstruction results (Fig. 4). However, the gold densities in these two models localize further away from the S-domain residues than observed in our experimental structure. Conversely, the gold densities in the third rigid modeling structure (Fig. 3c) do not form a ring but localize closer to the 5-fold S-domain residues. This could be explained by limitations in rigid modeling parameters that did not allow overlap between probe density (including organic ligand surface) and nanocapsid residues. Potential attractive forces between the C_6_MI linker and 5-fold surface that cause the AuNCs to partially overlap with the S- domain would not be accounted for in the rigid models. The rigid modeling results revealing a ring-shaped density around 5-fold center supported the experimental reconstruction data.

Ligand exchange bioconjugation was previously described using Au_144_ *p*MBA_60_^14,15^, a gold nanocluster similar to Au_102_. The surface thiol on Au_144_ *p*MBA_60_ was conjugated to a small Fab fragment that specifically bound a neuraminidase protomer. Single particle reconstruction results of these AuNC-protein complexes validated the specificity and immobility of AuNC-bound protein. These results suggested AuNC conjugation could be employed to optimize high-resolution cryoEM structure determination^16^. Further supporting the claim of AuNC-protein complex rigidity, we resolved the position of gold densities bound to the surface of nanocapsids at 12.8 Å resolution.

The high intensity regions in the nanocapsid 5-fold revealed in 3D reconstruction were not observed in either the unbound nanocapsid x-ray crystal structure or the reference density volume of T = 1 nanocapsid^29,35,36^, suggesting these intensities were attributable to bound AuNCs on the nanocapsid surface. The experimental measurements determined in 3D reconstruction were compatible with the size of AuNC and its maleimide linker (Fig. 4b,c). The atomic radius of Au_102_ is about 1.3 nm with a 1.1 nm C_6_MI linker arm^23^, such that, the distance from the center of the AuNC gold core to the maleimide conjugation site is approximately 2.5 nm. Similarly, when measured at 100% mass, the high intensity region attributed to AuNC in the 3D reconstruction was approximately 2.5 nm (Fig. 4b). A 2–3 nm distance between the maleimide binding site and the AuNC core density indicates the C6MI linkers extended from the binding site towards the 5-fold center without significant structural changes of the nanocapsid P-domain. The observation of intensities slightly above the S domain around the 5-fold center suggests bound AuNCs localized near neighboring AuNCs.

3D reconstruction with relaxed icosahedral symmetry indicated the AuNCs were not equally distributed around the nanocapsid 5-fold axes. The position of high intensities regions revealed in the relaxed symmetry structure was consistent with the imposed symmetry structure, but relaxed symmetry results suggested not all binding sites were occupied. These findings fit well with our SDS-PAGE and size exclusion results that indicated incomplete AuNC binding. Qualitative cryoEM observations also indicated AuNC conjugation was not homogenous among nanocapsids. The observed heterogeneity of AuNC binding to nanocapsid was distinct from previously described enterovirus binding results where only 0% or near 100% AuNC occupancy was observed^23^.

The nanocapsid 5-fold forms a sizeable cavity suitable for AuNCs with limited steric hindrance. The S-domain that forms the 5-fold axis is comprised of key hydrophobic residues and is relatively neutral in charge^25^, likely resulting in attractive forces between the S-domain 5-fold residues and C6MI linkers. The partial hydrophobicity of Au_102__C_6_MI likely contributed to attractive forces between neighboring AuNCs within the nanocapsid 5-fold. It was previously shown that, of the five cysteine replacement sites tested, 573 C functionalization with maleimide-biotin yielded the highest streptavidin binding signal^6^. It was proposed that 573 C conjugation resulted in multiple biotin conjugates localizing in the 5-fold, allowing the bound streptavidin to form its native tetrahedron structure above the 5-fold cavity. Our 3D reconstruction of AuNC-bound nanocapsids suggests that the 5-fold cavity is amenable to interactions between conjugate molecules without compromising the capsid structure.

AuNC conjugation optimization is critical to developing a stable, detectable delivery construct. A nanocapsid is conveniently sized to simultaneously anchor tissue-targeting ligands and detection molecules to the capsid surface^5^. AuNC detection molecules localized in the 5-fold cavity would not interfere with targeting molecules and/or foreign antigens chemically conjugated to the exposed dimer plateau. Nanocapsids were previously used to encapsulate delivery agents such as nucleic acids or negatively charged therapeutics^4,5,35^. This was achieved through reversible *in vitro* disassembly through chemical reduction and chelation, followed by the addition of negatively charged molecules and subsequent encapsulation via Ca^2+^ induced nanocapsid reassembly. This process exploits the Ca^2+^ salt bridges formed in the nanocapsid 3-fold that are critical to icosahedral assembly. By designing a linker and binding site that orients the AuNCs towards the 5-fold, AuNC conjugation would not impede packaging through 3-fold disassembly and reassembly. Importantly, establishing the 5-fold localization of conjugated AuNCs is conducive to high resolution tracking of nanocapsid packaging in cryoEM.

By resolving the structure of the AuNC-bound complex, an opportunity arises for higher order surface tuning with AuNCs. In a recent study^28^, a plasmonic AuNC, Au_~250_(*p*MBA)_x_ (Au_~250_), was linked through disulfide bonding to form dimer and trimer multimers. Au_~250_(*p*MBA)_x_ exhibits localized surface plasmonic resonance (LSPR) hallmarked by absorbance at 530 nm, which is considered a potentially useful signal for imaging biological samples^13,37^. Disulfide linkage between Au_~250_ resulted in plasmonic coupling in Au_~250_ monomers with unique UV-Vis absorbance at A_620 nm_ and A_800 nm_. This red-shift resulting from hybridized plasmonic behavior was greater than expected based experimental data with colloidal gold. Absorbance in red to near-infrared (NIR) range is a spectral window of minimum water absorbance and minimized scattering, ideal for “deep tissue” and/or *in vivo* imaging^37,38^. Nanocapsids could serve as a scaffold to orient similar plasmonic coupling interactions between AuNCs. As a diagnostic delivery system, a tumor targeted nanocapsid could be detected through LSPR and/or NIR detection. To control and optimize AuNC plasmonic coupling on the nanocapsid surface, it is critical to determine the position of AuNCs on the nanocapsid surface.

This study aided in the development of an efficient method for binding site selection, AuNC linker design, and AuNC conjugation to large icosahedral structures. The resolved structure of AuNC-bound nanocapsids offers insight for future ligand design, not only for additional conjugation, but also for coordination between bound AuNCs. Conjugation optimization and structural determination of AuNC-bound nanocapsids provided key insight in to the development of a theranostic nanocapsid delivery platform.

## Methods

### Nanocapsid Engineering and Production

Self-assembling nanocapsids were produced and purified following previously described methods^39^. Briefly, Hepatitis E Virus capsid protein sequence with 111 N-terminal and 52 C-terminal truncations (*dORF2)* was inserted in to the Baculovirus transfer vector (pFastBac/dORF2-HEV). N573 was replaced with cysteine using Phusion® Site-Directed Mutagenesis (Thermo Fisher Scientific) and the resulting pFastBac/dORF2-HEV_N573C plasmid was transformed and amplified in MAX Efficiency® DH5α™ Competent Cells (Thermo Fisher Scientific. Plasmids were purified with Qiagen Mini-Prep Kit and sequenced at the UC Davis Sequencing Facility.

Following the Bac-to-Bac® Baculovirus Expression System protocol, pFBac/dORF-HEV_573 C plasmids were subsequently transposed in to MAX Efficiency® DH10Bac™ Competent Cells and screened twice for insertion in to the Bacmid vector (Bacmid/dORF2-HEV_573 C). Following the manufacturer’s protocol, purified Bacmid/dORF2-HEV_573 C was transfected in to SF9 cells from the Izumiya lab for production of baculovirus recombined with dORF2-HEV_573 C. Five-seven days after transfection, SF9*/*baculovirus supernatant was passaged two times in SF9 cells and collected three days post infection for baculovirus amplification.

A suspension culture of High Five insect cells was infected with amplified baculovirus supernatant for the production of HEV nanocapsids. High five cells expressing nanocapsid were harvested six days post infection. Nanocapsids were isolated from supernatant with previously described methods, using sucrose and cesium chloride gradient purification and NP-40 detergent treatment to remove lipids. SDS-PAGE was used to screen fractions for purity and presence of nanocapsid depicted by 52 kDa banding. The storage and imaging buffer for nanocapsids was 10 mM MES pH = 6.2. Nanocapsids were imaged in TEM to confirm an intact T = 1 icosohedral structure was formed. The presence and exposure of nanocapsid cysteine was confirmed as previously described^6^.

### Au_102_(*p*MBA)_44_ and Au_102_ C6MI Synthesis and Purifcation

Au_102_(*p*MBA)_44_ (Au_102_) was synthesized using previous methods^17,40^. In this work N-(6-hydroxyhexyl)maleimide was covalently bound via Steiglich esterification reaction with p-mercaptobenzoic acid (*p*MBA) protected Au_102_ cluster to form Au_102__C_6_MI^23^. Synthesis was done in dry DMSO/DCM in the presence of dicyclohexylcarbodiimide (DCC). The amount of linker molecules per cluster cannot be accurately controlled but it was kept sufficiently low so that the functionalized clusters remained water-soluble.

Gel electrophoresis was run on 15% polyacrylamide gel (29:1, acrylamide:bisacrylamide) using 2X TBE buffer in a Bio-Rad Mini-Protean Tetra System gel electrophoresis apparatus at 130 V for two hours. All materials were commercial unless otherwise mentioned and used without further purification. Both Au_102_ and Au_102__C_6_MI AuNCs were stored in dry conditions at RT or at 4 °C before conjugation. Specifically, Au_102__C6MI was only solubilized in a neutral pH solution immediately before conjugation to avoid the loss of maleimide function through hydrolysis.

### Nanocapsid and AuNC Pre-Conjugation Preparation

AuNCs were diluted in sterile ddH_2_0, 20 mM Tris-HCl/150 mM NaCl, PBS, or 10 mM MES. In the case that AuNC precipitate formed in buffer, serial pelletation and resuspension was carried out to isolate soluble Au_102__C_6_MI. The concentration of nanocapsids was determined with spectrophotometric absorbance measurements at A_280nm_ followed by molar conversions based on the previously published molecular weight of T = 1 HEV_LP^35^. The concentration of solubilized AuNCs were determined with modified calculations based on previously described Au_102_ extinction coefficients at A_420nm_^14,20^.

Prior to conjugation, nanocapsids were diluted 1:1 nanocapsid:neutral buffer (neutral buffer was either Tris-HCl/150 mM NaCl pH = 7.5 or PBS) and 1 mM TCEP final concentration. Nanocapsids in neutral buffer/1 mM TCEP were incubated at RT for 30 minutes-1 hour.

### Nanocapsid Conjugation to AuNCs and Purification

Conjugation carried out 5 h or ON at RT or 4 °C. Gravity column chromatography and fractionation was used to separate unbound AuNC from nanocapsid-conjugated AuNC. Approximately 0.5–1 mL of GE Healthcare Sephacryl^®^ S-500 HR size exclusion chromatography resin was serially washed with nanocapsid buffer (10 mM MES) and placed in Biorad Poly-Prep^®^ Chromatography Columns pre-calibrated with sample buffer. In the column, the resin was serially washed and packed to form flat resin surface. Each droplet from the gravity column was separately collected such that each Eppendorf contained the same volume (35 μL). As a gravity-based method, time intervals between droplets was consistent over the course of collection. Individual fractions were assessed by spectrophotometry measurements and visible color change. Those fractions with peak A_280nm_ and/or A_420nm_ readings were screened with gel electrophoresis, and TEM imaging. Unbound AuNC remained in the column while nanocapsid and nanocapsid-bound AuNC eluted from column. Fractions were handled at 4 °C.

#### Spectrophotometry

For AuNC-bound nanocapsid samples, AuNC and nanocapsid concentration were determined by combining previously described calculation methods^14^ and Au_102_-specific extinction coefficient calculations^20^.

A_420nm_ measurements were used indicate the concentration AuNC in the sample. A_420nm_ was then used to infer the expected A_280nm_ value based on known UV-Vis absorption energies^20^.

1 $$\begin{array}{rcl}{\rm{AuNC}}\,{\rm{concentration}} & = & {{\rm{A}}}_{420{\rm{nm}}}/33338.439\,\mathrm{mol}/L\\ {\rm{Expected}}\,{{\rm{A}}}_{280{\rm{nm}}}\,{\rm{of}}\,{\rm{AuNC}} & = & {{\rm{A}}}_{420{\rm{nm}}}\times 2.072\\ {{\rm{A}}}_{280{\rm{nm}}}\,{\rm{of}}\,{\rm{nanocapsid}} & = & \begin{array}{c}{(\mathrm{Measured\; A}}_{280{\rm{nm}}}\,{\rm{of}}\,{\rm{AuNC}}\\ +\,{\rm{nanocapsid}}-{\rm{Expected}}\,{{\rm{A}}}_{280{\rm{nm}}}\,{\rm{of}}\,{\rm{AuNC}})\end{array}\end{array}$$

### SDS-PAGE

Nanocapsid alone was run in reducing SDS-PAGE while samples containing AuNC was run in non-reducing SDS-PAGE. AuNC samples were prepared in non-reducing SDS loading dye (without β-mercaptoethanol or DTT) to protect the AuNC core from damage as previously described^14^. 52 kDa banding indicated the presence of nanocapsid, while a band ~56 kDa and visibly brown pre-coomassie blue staining indicated AuNC-bound nanocapsid.

#### TEM

Samples were added to ionized continuous carbon grids, washed briefly with ddH20, and negatively stained with 2% uranyl acetate. Grids were then imaged on JEOL1230 Transmission Electron Microscope to confirm stability of nanocapsid structure^35^.

### CryoEM Preparation and Imaging

CryoEM preparation and imaging was carried out using previously described methods^35,41^. Briefly, homemade lacey carbon coated grids and 200-Mesh Quantifoil^®^ holey cabon grids were ionized with 40 mA glow discharge for 30 seconds. 1–2 mg/mL sample was added to the ionized grid for 30 seconds, blotted, and immediately plunged in to liquid nitrogen cooled liquid ethane using both manual and Virtobot^®^ cryo-plunging methods. Nanocapsids embedded in a thin layer of vitrified ice were transferred to JEOL JEM-2300 microscope specimen chamber using a Gatan 626 cryo-transferring system. The sample was imaged with TVIPS CCD camera (TemCam-F415) under 60,000x magnification with a recorded pixel size of 1.667 Å. The specimen was imaged with between 0.5–4 Å defocus level. The total dosage per image was approximately 60 electrons/ Å^2^. Alternatively, defocus pairs were imaged at 30 electrons/ Å^2^ dosage.

### Rigid Modeling

To model Au_102_, a radial distribution function of atoms was generated using Gaussian distribution of 0.1 Å broadening parameter and the DFT-relaxed Au_102_(*p*MBA)_44_ coordinates. A simplified electron density of a single Au_102_ was generated using 10 Gaussians that match each of the atomic shell of the true radial distribution function. 7 Gaussians were used for Au-atoms, 1 for S-atoms, 1 for C-atoms, and 1 for O-atoms. H-atoms were neglected. Cumulatively, several thousands of possible Au_102_ positions was found and modeled electron density of a single Au_102_ was added to the allowed Au_102_-positions to generate the overall modeled electron density of Au_102_ bound to the CYS-sites around a single 5-fold symmetry axis of nanocapsid. This kind of density describes how the electron density of Au_102_ nanoparticles that are bound to the CYS-sites should look on average. While probe densities could not overlap with nanocapsid coordinates, the parameters required some point on the probe density was in contact with some point on the nanocapsid coordinates.

### Reconstruction

From 113 micrographs, a total of 5276 raw projection images were selected via semi-automated particle selection tools using EMAN 2.1 package^42^. The selected particles were then screened manually and further segregated for quality by real space cross-correlation (CC) analysis from a set of projection images obtained from x-ray model^36^. After 7 cycles of refinement, particles with highest cross correlation values (CC), based on normal distribution were separated for the final 3D reconstruction. CC of + /− 20%, + /− 15%, + /− 5% were progressively used to screen particles. Approximately 33% of selected particles were used in the final reconstruction. Progressively, during these refinement cycles, particles with + 5% RMSE of both center and orientation were excluded from the 3D reconstruction. The screened particles were further separated into subpopulations according to estimated diameter through scaling factor measurements, using the Polar Fourier Transformation (PFT) methods^31^. The population with the highest CC value and lowest root mean square error (RMSE) in size variation were then low-passed to 20 Å to reduce background noise and were used to generate a low-resolution initial model though EMAN 1.9’s StartIcos program^43^. The refinement loop was optimized through a number of steps, gradually relaxing the low pass, verified by increased overall convergence. Furthermore, the orientation and center assignments for individual particles were carefully screened over the span of six cycles through the PFT method. A final radial search was conducted to isolate highly representative projection images, possessing high intensity peaks, most likely representing clusters of Au102 gold nanoparticles. The final resolution of the map was determined by Fourier Shell Correlation (FSC) via the even/odd split test via JSPR package^32^, estimated to 12.8 Å.

## Electronic supplementary material

Supplementary information
