# Supplementary material for: Structural characterization of site-modified nanocapsid with monodispersed gold clusters
Source: Sci Rep. 2017 Dec 6;7:17048. doi: 10.1038/s41598-017-17171-x (PMC5719084; doi:10.1038/s41598-017-17171-x)
Supplement: Supplementary file 1 — Supplementary information [file 41598_2017_17171_MOESM1_ESM.pdf]

Structural characterization of site-modified nanocapsid with monodispersed gold clusters

Marie C Stark[1,2], Mo A Baikoghli[2], Tanja Lahtinen[3], Sami Malola[4], Li Xing[1], Michelle Nguyen[2], Marina Nguyen[2], Aria Sikaroudi[2], Varpu Marjomäki[1], Hannu Häkkinen[3,4*], R Holland Cheng [1,2*]

Supplementary Data

Supplementary Figure 1

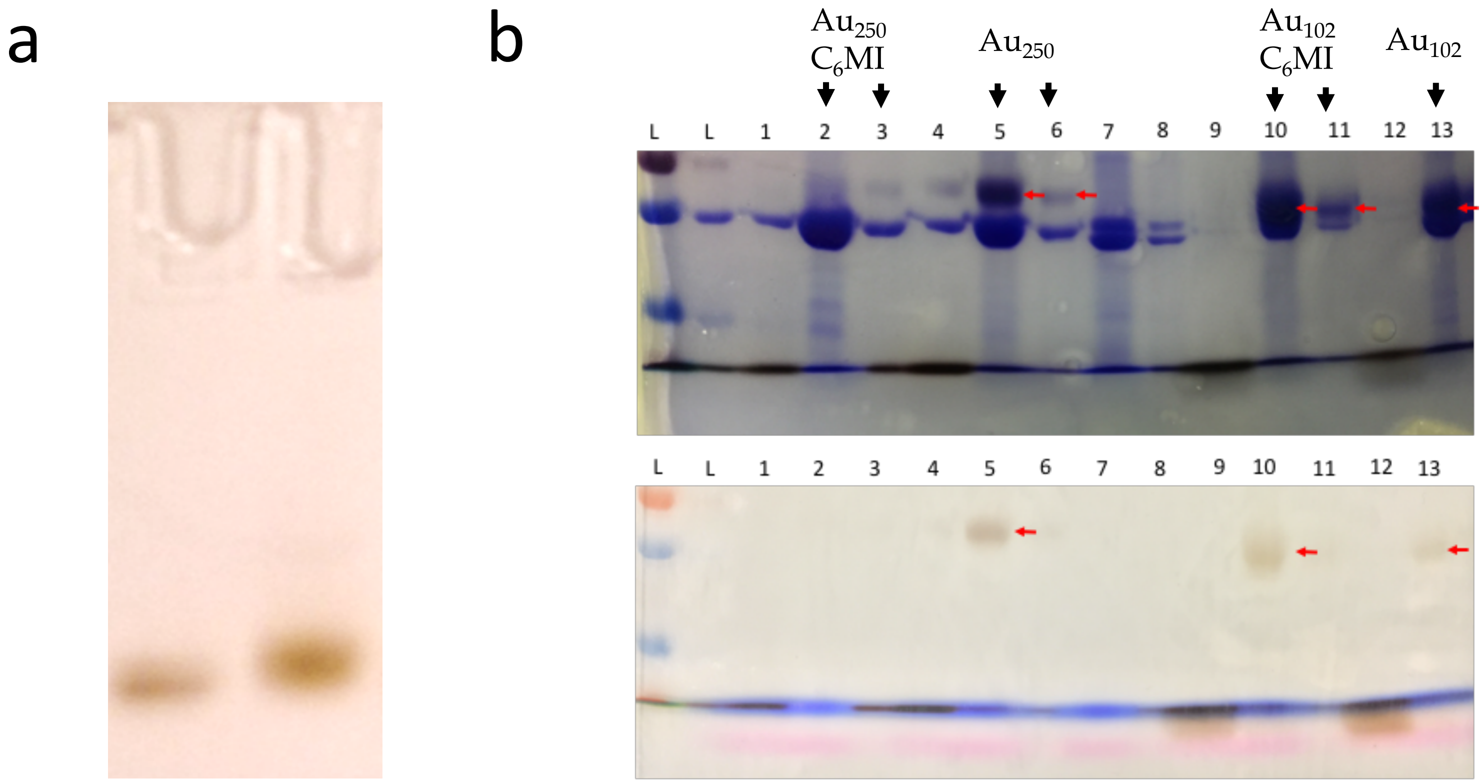

Full PAGE Gels (a) Native PAGE gel of Au102 (left) and Au102_C6MI (right). (b) SDS-PAGE gel of nanocapsids bound to different gold

Supplementary Figure 2

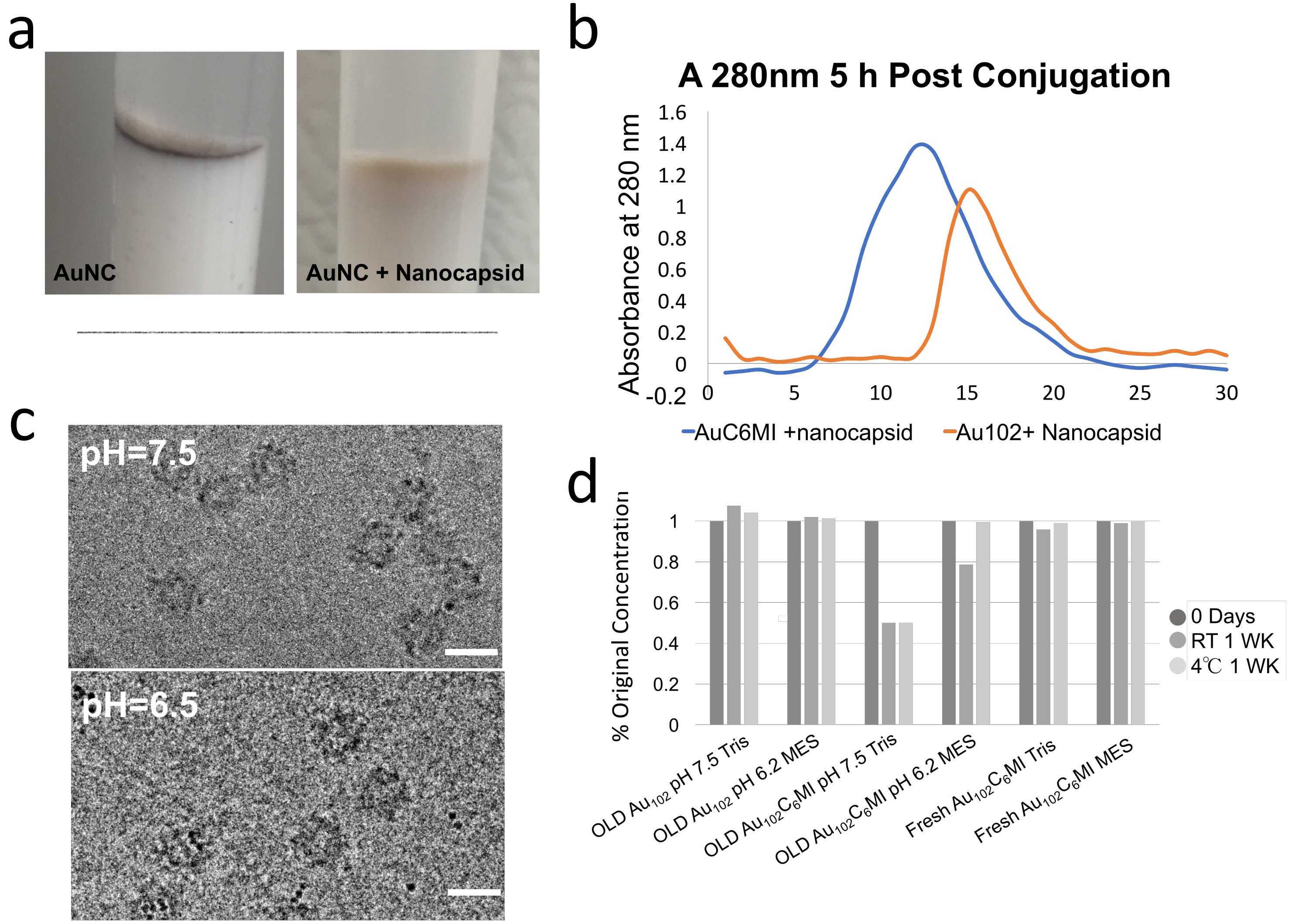

AuNC-bound nanocapsid purification and AuNC precipitation (a) Size exclusion chromatography resin of unbound AuNC and AuNC bound to nanocapsid (left). (b) Nanodrop of size exclusion chromatography fractions 5h post-conjugation for Au_{102}_C_6MI -bound nanocapsid (blue) and Au_{102}-bound nanocapsid (red). (c) Differences in distribution of Au_{102}_C_6MI bound nanocapsids in Tris-NaCl (pH=7.5) and MES (pH=6.5). (d) Concentration changes over time of unbound Au_{102} and Au_{102}_C_6MI in Tris-NaCl (pH=7.5) and MES (pH=6.5) buffers. Reduced concentration percentage indicates precipitation in solution.

Supplementary Figure 3

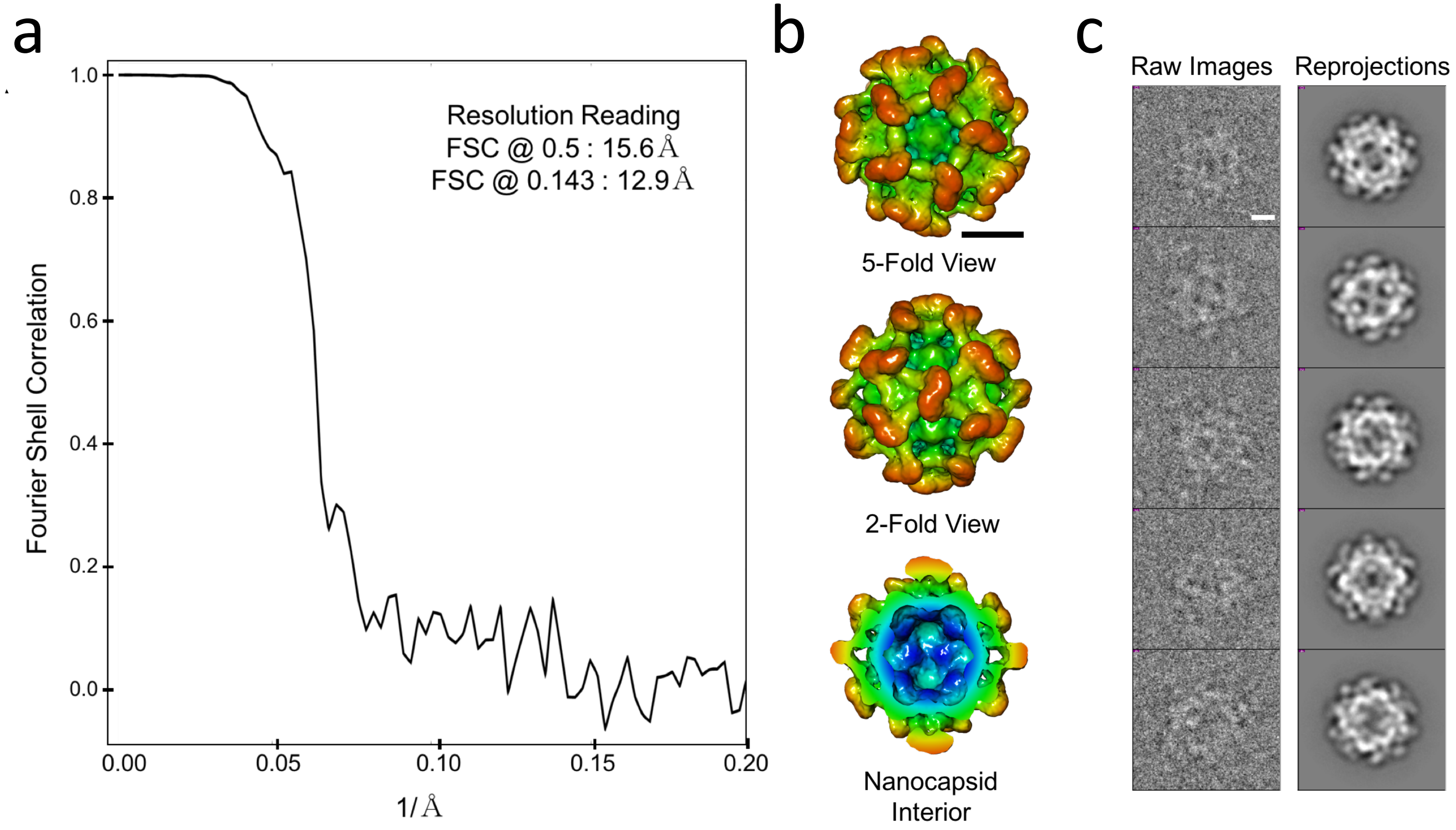

3D reconstruction results (a) Fourier Shell Correlation resolution evaluation reported at 0.143 and 15.6 Angstroms at 0.5. (b) 3D rendering of AuNC-bound nanocapsid shown at 5-fold, 2-fold, and interior views. Radial coloring was employed to illustrate S, M, and P domain with gradient coloring of blue, green, and orange, respectively. (c) Raw projection images of selected particles and 3D projection images show strong correlation. Scale bar: 10 nm.

Supplementary Figure 4

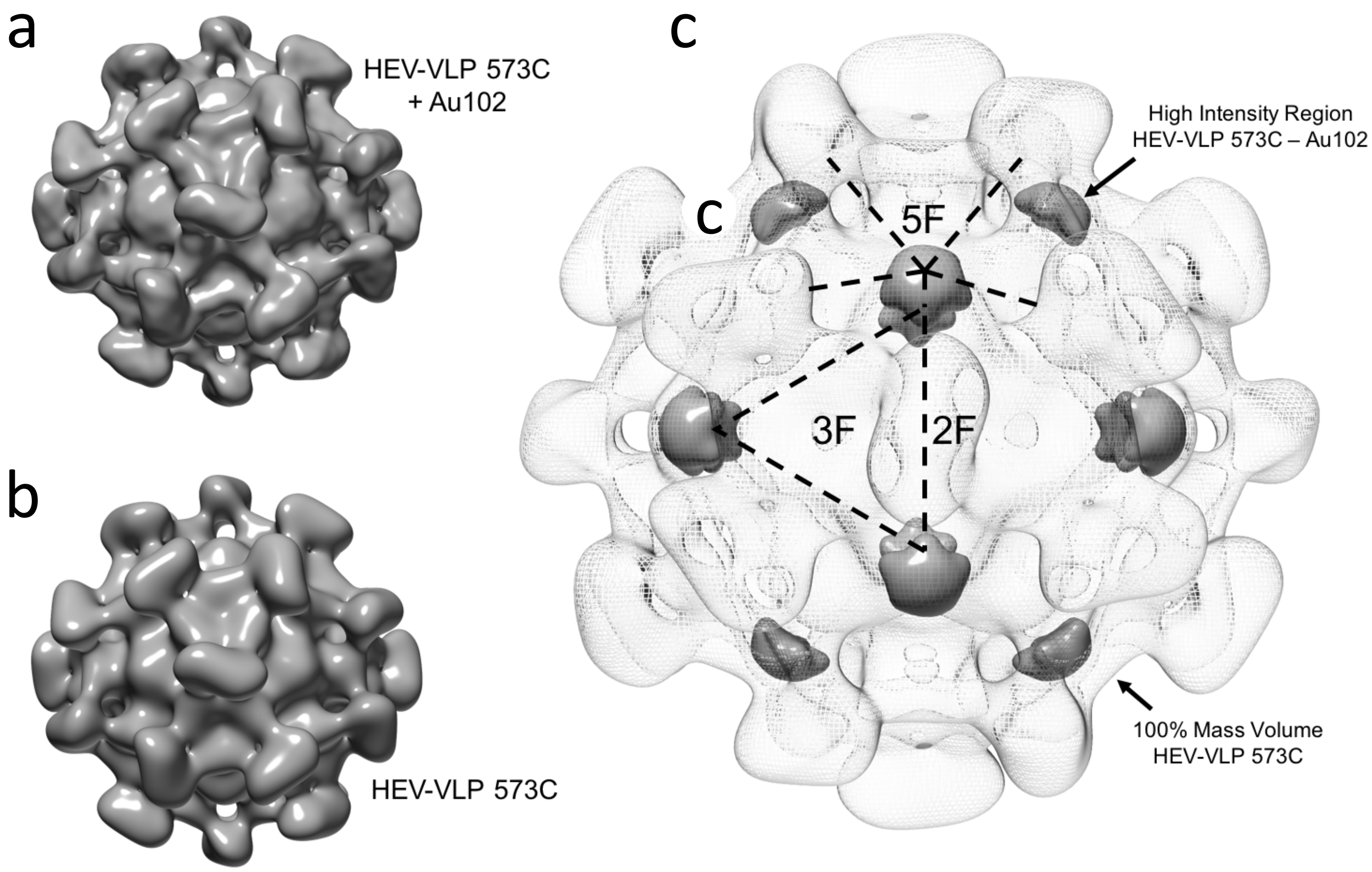

Difference mapping (a) 3D rendering of AuNC-bound nanocapsid and (B) unbound nanocapsid. Difference mapping illustrates novel high intensity regions formed around the 5-Fold axis. For comparative purposes, both density maps were low-pass filtered to 20 Angstroms. Scale bar: 10 nm.
